# Qualitative insights into patient and carer perspectives of malignant pleural effusion management: an embedded study within the TACTIC trial

**DOI:** 10.1136/bmjresp-2026-004098

**Published:** 2026-07-27

**Authors:** Alice Milne, Alexandra Dipper, Rahul Bhatnagar, Anand Sundaralingam, Najib M Rahman, Nick Maskell, Andrew J Moore

**Affiliations:** 1 Academic Respiratory Unit, University of Bristol, Bristol, UK; 2 Oxford University Hospitals NHS Foundation Trust, Oxford, UK; 3 Oxford Respiratory Trials Unit, University of Oxford, Oxford, UK; 4 Musculoskeletal Research Unit, University of Bristol, Bristol, UK

**Keywords:** Pleural Disease, Mesothelioma, Lung Cancer, Patient Outcome Assessment

## Abstract

**Background:**

Malignant pleural effusion (MPE) is a condition characterised by excessive fluid build-up in the pleural cavity caused by cancer. Management options focus on drainage of fluid from the pleural cavity with a common aim of minimising symptoms and improving quality of life. Definitive pleural intervention is recommended for most patients with MPE to achieve longer-term symptom control. A patient-centred approach is also emphasised. However, little is known about the patient and carer experiences of MPE management. Therefore, this study sought to examine patient and carer experiences of differing procedural interventions within a trial setting.

**Methods:**

Patients with MPE and their carers who were taking part in the TACTIC trial in hospitals across the UK were invited to take part in a one-off interview. Thirty-two participants were recruited, and 22 semistructured interviews were conducted. Interview formats were either patient only, carer only or patient and carer dyad interviews. Interviews were audio recorded, transcribed verbatim and analysed using a generalised thematic analysis approach.

**Results:**

Initial findings demonstrated that patients and carers were generally satisfied with the MPE management and care received. No significant differences in acceptability were found between the two management strategies. Further analysis led to the development of four overarching themes: experiences of MPE beyond the trial, experiences of pain, preferences in MPE care and measuring MPE: rethinking patient relevance. These findings underscore the importance of greater consideration of pain management during MPE care, as well as improved communication and information prior to procedures.

**Conclusion:**

Patients and carers found the TACTIC combined procedure tolerable and acceptable. Echoing the growing evidence of research in this field, authors emphasise the importance of outcome measure selection in MPE trials and suggest a need for a Core Outcome Set to establish fundamental priorities in MPE management.

**WHAT IS ALREADY KNOWN ON THIS TOPIC**

- There are multiple interventions available for the management of malignant pleural effusions (MPE) supported by high-quality clinical trial findings. Here, we explore the experience of MPE management from the patient and carer perspectives and examine the challenges associated.

**WHAT THIS STUDY ADDS**

- This study addresses an important gap in the current literature regarding the limited understanding of the perspectives of patients and their carers. Insights are provided into how acute pain is managed and communicated to patients diagnosed with MPE.

**HOW THIS STUDY MIGHT AFFECT RESEARCH, PRACTICE OR POLICY**

- The relevance and utility of outcome measures currently used in pleural trials are called into question, underscoring the need to re-evaluate MPE outcomes to inform patient-centred clinical trial design and future practice.

## Introduction

### Malignant pleural effusion

 Malignant pleural effusion (MPE) affects an estimated 50 000 people in the UK each year.^1^ It is a condition characterised by excessive fluid build-up in the pleural cavity caused by either mesothelioma (primary pleural malignancy) or a spread of cancer from a secondary site. The most common causes of MPE are metastatic lung cancer in men and breast cancer in women.^2^ Although prognosis is variable, survival duration is usually between 3 and 12 months.^3^ Patients with MPE often experience severe breathlessness, which can impact on quality of life and reduce daily physical activity.^4^ Management options for MPE focus on drainage of fluid from the pleural cavity. The differing procedural interventions have a common aim of minimising symptoms and improving quality of life. British Thoracic Society Guidelines^5^ advise definitive pleural intervention for most patients with confirmed MPE with the aim of achieving longer-term symptom control, with either an inpatient admission for talc pleurodesis (administered as slurry through a chest drain or poudrage during medical thoracoscopy) or an indwelling pleural catheter (IPC) inserted with a day case procedure. Guidelines also emphasise a patient-centred approach to MPE management, accounting for individual preferences and treatment priorities when deciding on a management strategy.

### Current evidence on patient perspectives

Current literature on the patient experience of different MPE interventions and the views of those who care for them is limited.

Twose *et al*^6^ explored the impact of therapeutic thoracentesis (TT) on a wide range of symptoms. Through interview data, the study demonstrated improvements in patient-reported sleep post-TT but no improvement to low appetite, anxiety or fatigue. In support of these findings, Aujayeb and Wakefield recommended^7^ that future research should consider how MPE interventional strategies influence a wider range of symptoms experienced by patients, beyond breathlessness. Indeed, itching, difficulty sleeping and the IPC as a reminder of the patient’s disease were highlighted as predominant issues in a survey of patients receiving an IPC.^8^

Building on this, findings from a single-centre qualitative study exploring patients’ experiences of living with an IPC^9 10^ demonstrated a negative impact on engagement in activities, independence and anxiety (interviews conducted at 2 weeks and 6–8 weeks post-IPC insertion). The level of impact of these factors on patients was found to be influenced by existing social support, quality of care and overall cancer impact.^10^ Positive impacts included reassurance from the presence of an IPC in situ.

The only qualitative study to date to examine patient experience of talc pleurodesis^11^ is also a single centre interview study. The study highlighted a lack of consensus among patients regarding a preferred MPE management route, emphasising the importance of informed decision-making.

Symptom control is a key priority for patients^12^ and differing interventions for MPE can now be adapted and combined to meet individual treatment priorities.^13–15^ It is essential therefore that as procedural practice evolves, our understanding of the patient experience keeps pace to enable informed and relevant discussion when deciding on a management strategy.

### Purpose of this study

This study aimed to examine patient and carer experiences of medical thoracoscopy and talc pleurodesis (TTP) with or without IPC insertion within a trial setting.

## Methods

### Study design

Here, we report findings from a qualitative sub-study embedded within the multicentre TACTIC phase III RCT^16^ (National Institute for Health Research PB-PG-1217-20037).

Trial participants had symptomatic MPE and were randomised to receive either a combined procedure with medical thoracoscopy, talc pleurodesis and IPC insertion (TTP+IPC) or standard care with TTP alone (see online supplemental appendix A). Co-primary outcomes of the trial were breathlessness and hospital length of stay over 4 weeks post procedure. Trial findings will be reported separately. All TACTIC participants (from 11 participating NHS hospital sites across the UK) received information about this qualitative study from local research teams. One-off semistructured telephone interviews were conducted with patients and carers, to explore their experience of MPE management. We have reported the findings here in line with COREQ guidelines^17^ (online supplemental appendix B). The topics covered in the interviews were detailed within the information sheets provided, and the topic guides were carefully reviewed by three PPI members. Informed consent was recorded at the beginning of the interview and re-confirmed at its culmination. A distress protocol was designed to be used in the event of a participant becoming upset.^18^

### Interview participants

Eligible patients were adults, had suspected or confirmed symptomatic MPE (as defined in the TACTIC protocol^16^) or were a carer for someone with MPE. Carers were defined as a family member or close friend who regularly supported the patient. Thirty-two participants were recruited from eight hospital sites across the UK (out of the 11 sites participating in the TACTIC trial), including 21 patients (five female and 16 male) and 11 carers (six female and five male) (see table 1). All names included are pseudonyms. The age of patients ranged from 41 to 86, with a median age of 73. Eleven interviews were patient only, 10 were patient-carer dyads and one was carer only.

**Table 1:** Patient characteristics

Pseudonym	Gender	Age (years)	Primary cancer	TACTIC trial randomisation
Gillian	Female	76–80	Mesothelioma	TTP+IPC
Carol	Female	66–70	Mesothelioma	TTP+IPC
Colin	Male	76–80	Mesothelioma	TTP+IPC
Judith	Female	81–85	Breast	TTP+IPC
Brian	Male	86–90	Mesothelioma	TTP+IPC
Russell	Male	61–65	Mesothelioma	TTP
Florence	Female	71–75	Other	TTP+IPC
Thomas	Male	71–75	Lung	TTP
Frank	Male	81–85	Mesothelioma	TTP+IPC
Arthur	Male	71–75	Lung	TTP+IPC
Harry	Male	61–65	Lung	TTP
Keith	Male	81–85	Mesothelioma	TTP
Stephen	Male	71–75	Mesothelioma	TTP+IPC
Stuart	Male	66–70	Mesothelioma	TTP+IPC
Alan	Male	76–80	Mesothelioma	TTP
Derek	Male	66–70	Lung	TTP
Joshua	Male	66–70	Mesothelioma	TTP+IPC
William	Male	71–75	Unknown	TTP
Neil	Male	76–80	Lung	TTP
Francis	Male	76–80	Mesothelioma	TTP+IPC
Connie	Female	41–45	Unknown	TTP

IPC, indwelling pleural catheter; TTP, medical thoracoscopy and talc pleurodesis.

### Data collection

TACTIC trial participants who confirmed interest in the study, provided consent to be contacted by a qualitative researcher (AM), who organised a suitable time and date for the interview. This was also an opportunity for participants to ask any questions about the interview.

Twenty-two interviews were conducted between 4 and 16 weeks post-procedure, allowing for flexibility for participants, and were chosen to avoid overburdening participants. Sample size was guided by information power.^19^ Interviews were conducted by the lead author AM (Milne), an experienced qualitative health researcher and AD (Dipper), a pleural clinician and clinical trialist, establishing investigator triangulation^20^ (both female). Field notes were made after each interview. Post-interview debrief discussions were held with the research team. Three arranged interviews could not be conducted due to the patient feeling too unwell or being hospitalised.

AM had no relationship to any of the participants prior to this study. AD worked in a clinical capacity at one of the participating hospital sites during the trial. To ensure participants interviewed by AD were not known to her in a clinical capacity, these interviews were conducted with participants from external hospital sites only, whom she had no prior contact with.

Topic guides were piloted in the first two interviews. The wording was adapted to improve comprehension and flow; this alteration included clarification of the ‘trial procedure’ to clearly distinguish between any other procedures the patient may have had and to avoid ambiguity.

### Analysis

Audio recordings were transcribed, anonymised, and imported into QSR NVivo data management software. A generalised thematic analysis approach was adopted.^21 22^ This followed the five main phases outlined by Jowsey *et al*: ‘familiarising the researcher with the data’, ‘generating initial codes’, ‘searching for themes’, ‘reviewing the themes’ and ‘naming the themes’.^22^ Rigorous conduct was maintained throughout,^23^ with logs kept of each step of the analysis. Field notes were re-visited during familiarisation with transcripts, concurrent with continuing data collection. Recruitment stopped when it was agreed within the team that there was a high quality of dialogue and sufficient coverage of the specified research aims to attain information power.^19^

AM generated initial codes and independent double-coding was conducted by AD. Initial coding and theme generation aimed to examine experiences within the trial context by comparing the accounts of those who received the intervention with those who received standard care. Following initial coding, it became evident that the themes transcended the randomisation divide and were applicable to the broader experience of MPE management. The data were then revisited, and further analysis took a whole data approach considering the broader experiences of MPE across the data set (online supplemental appendix C). During this, AM and AD conducted an in-depth review of codes, prompting further examination of the data and theme review. Additional reflective discussions were then held with the wider team including AJM (Moore) (PhD, experienced qualitative methodologist), as well as clinical experts RB and NM. Interim findings were also shared and reviewed by the TACTIC trial steering committee.

## Results

We will present our results in two distinct sections. First, we describe our findings in the context of trial participation. Second, we will present four overarching themes constructed from a whole data approach of the wider experience of MPE management from the perspective of patients and their carers.

### Experience of MPE management within a clinical trial setting

The objective of this study was to examine patient and carer experiences of MPE management, including their experience of the combined procedure (TTP+IPC), within a clinical trial context. Patients randomised to receive TTP+IPC reported that they were very satisfied with their management. This was evident in their discussion of the experience of the procedure and the positive health impact they experienced post-procedure (table 2).

**Table 2:** Exemplary quotes

“the actual procedure and having the drain in wasn’t a problem’ (Gillian, patient, TTP+IPC)	‘I don’t remember feeling any pain, anything like that at all (…) I would advise it definitely yeah, it wasn’t uncomfortable, I didn’t feel any reason to be that concerned when it was there’ (Florence, patient, TTP+IPC)
‘It was all new to me so I just wanted to be better’ (Francis, patient, TTP+IPC)	‘I’m breathing better. I feel better. I’m walking better. I’m doing everything better’ (Brian, patient, TTP+IPC)
‘The impact has been that she’s come back better than when she went in (…) Before she had the procedure, she couldn’t make the top of the stairs at home without gasping. Having had the procedure, well, you just can’t stop her now. She’s like a racehorse’ (Graham, carer, TTP+IPC)	‘A good drain equals better quality of life. Because we could see the difference between before the first drain when I was very puffed, difficulty breathing and the cough and it improved immensely after that first big drain’ (Carol, patient, TTP+IPC)

IPC, indwelling pleural catheter; TTP, medical thoracoscopy and talc pleurodesis.

Patients randomised to TTP were also satisfied with their management. Participants viewed their involvement in the TACTIC trial positively, as expected in a self-selecting population. Participants in the TACTIC trial received increased clinical contact due to research follow-up schedules. The regular communication with pleural research nurses afforded by trial participation was welcomed and reassuring to patients.

Overall, regardless of which trial arm patients were randomised to, they reported being satisfied with the care they received and their experience of trial participation. Analysis of patient and carer accounts showed that both groups found their treatment acceptable.

### Experiences of MPE beyond the trial

**Figure 1:**
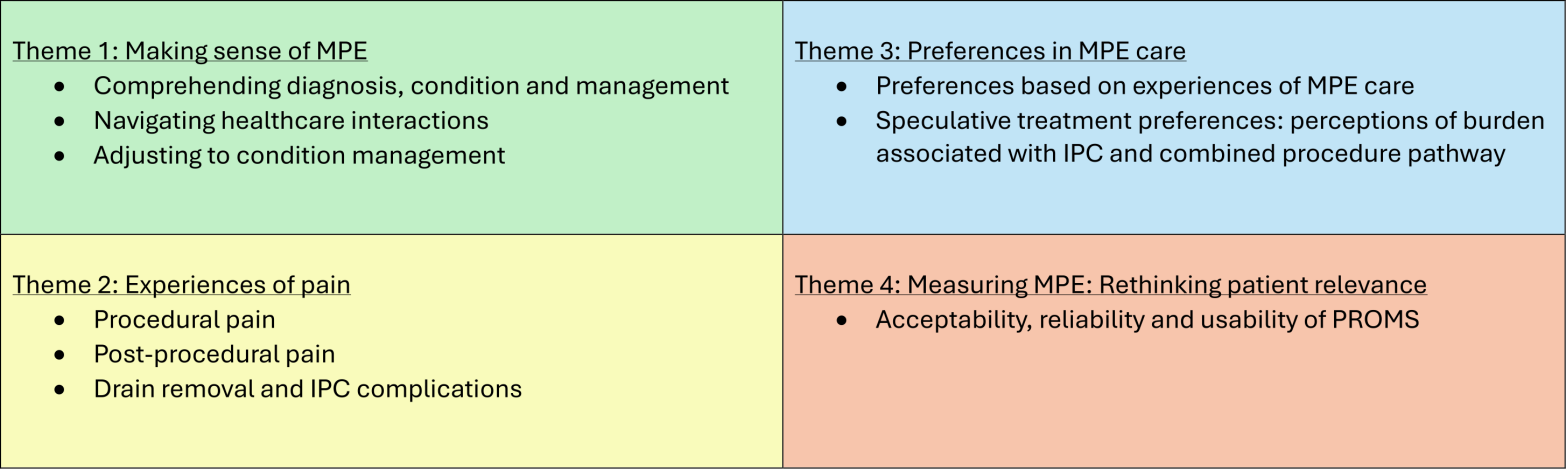
Table of themes. For quotes, see figures 2 and 3. IPC, indwelling pleural catheter; MPE, malignant pleural effusion; PROMs, patient-reported outcome measures.

During the second phase of analysis, we focused on the patient and carer experiences of MPE management beyond the context of the trial. Four themes were constructed (see figure 1).

**Figure 2:**
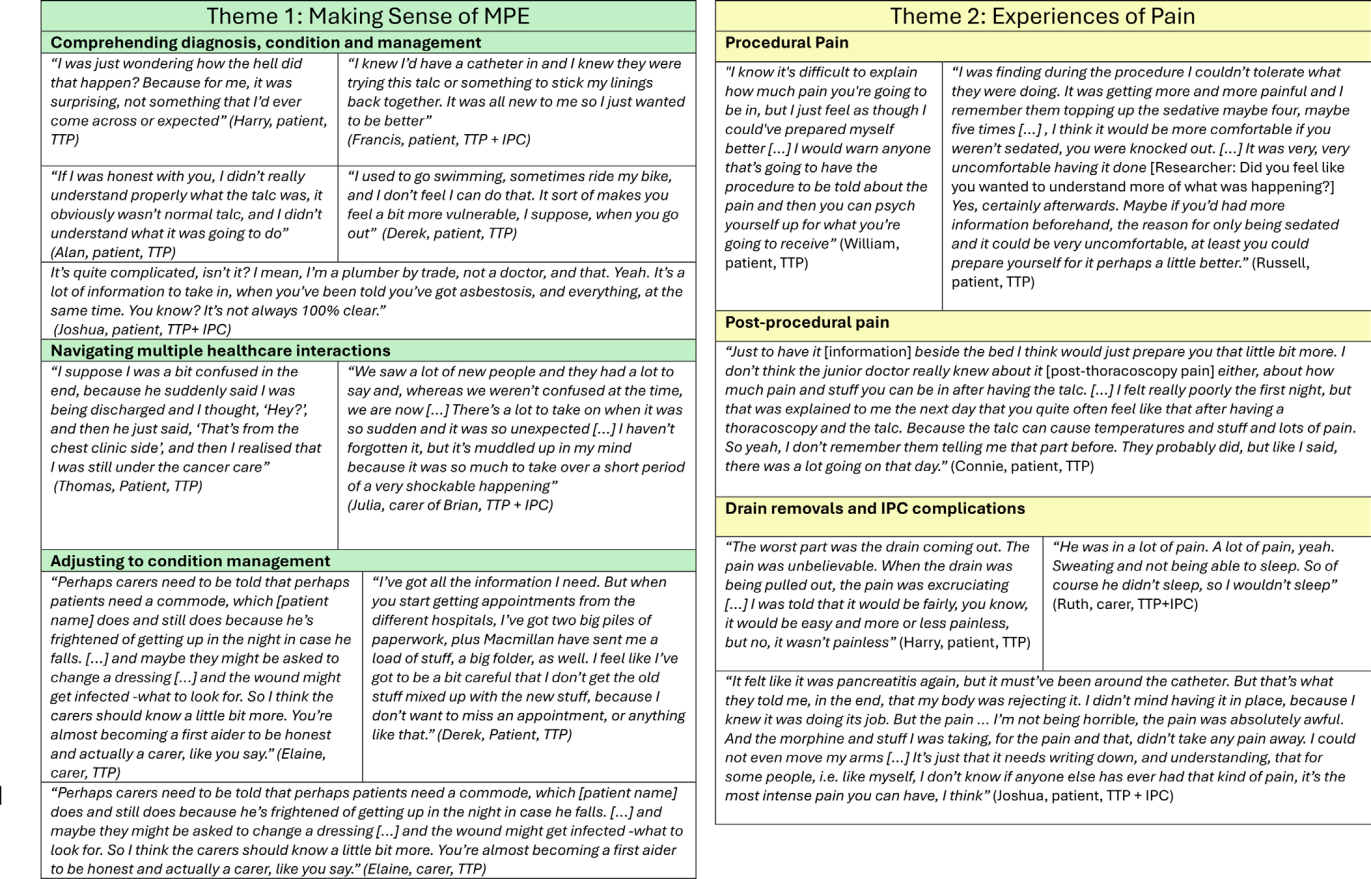
Quotations from themes 1 and 2. IPC, indwelling pleural catheter; MPE, malignant pleural effusion; TTP, medical thoracoscopy and talc pleurodesis.

**Figure 3:**
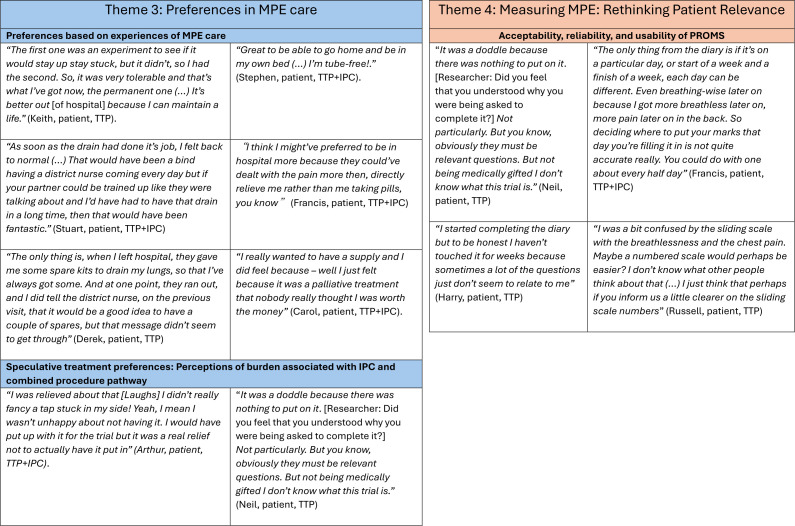
Quotations from themes 3 and 4. IPC, indwelling pleural catheter; MPE, malignant pleural effusion; TTP, medical thoracoscopy and talc pleurodesis.

#### Making sense of MPE

This theme captures the various ways in which participants made sense of their condition, including efforts to understand the diagnosis, navigate healthcare interactions, and adapt to managing the condition in daily life.

##### Comprehending diagnosis, condition and management

Prior to their diagnosis, many participants had no prior knowledge of MPE as a health condition associated with cancer. Some reflected on gaps in their understanding of the condition and subsequent management. The pathophysiology of MPE and mechanism for fluid build-up was challenging for patients to understand, as well the process of fluid removal, use of talc, and concept of pleurodesis.

Patients described a rapid change in their health status, the pace of which often intensified the struggle to make sense of MPE. Many participants expressed difficulty in coming to terms with this rapid deterioration, describing both a physical and psychological adjustment.

Patients and carers both described a sense of being overwhelmed, with some referring to this period as having a sense of finality—‘you felt your life was over*’* (Carol, patient, TTP+IPC).

##### Navigating multiple healthcare interactions

Participants described an influx of hospital visits and communication in the early period following diagnosis.

Russell (patient, TTP) reflected that ‘everything went crazy all at once’, while Diane (carer, TTP+IPC) used the metaphor of ‘snowballing’ to convey the accelerating momentum of a plethora of appointments received from various teams in a short period.

Patients and carers described how the increasing medical interaction made it difficult to distinguish between the roles of pleural physicians and oncologists. Some expressed confusion stemming from this dual management and the differing timelines.

##### Adjusting to condition management

Carers played an important role in helping patients to manage their MPE at home, and practical support was particularly valued. One patient living alone described the management of appointment schedules and expressed concern about missing or confusing them.

Many carers took on organisational responsibilities to ease the burden on patients, including recording and tracking clinical information, and some wanted better information to prepare them for their role. Elaine (carer) felt unprepared to support her husband’s hospital discharge and ongoing care, strongly advocating for improved communication and support for carers.

Nurse support was also valued by both carers and patients who frequently expressed gratitude for their sensitivity and practical support (e.g, arranging a disabled parking badge), emphasising the value of logistical assistance.

### Experiences of pain

Individual experiences of pain and pain management varied. For those who did experience pain, many expressed a desire for increased preparation.

#### Procedural pain

While most participants were relieved by the lack of procedural pain during TTP, some described having significant pain that they did not feel prepared for. For patients who received a non-diagnostic aspiration prior to TACTIC trial participation, a thoracoscopic pleural biopsy may have been conducted within their trial procedure. Although not the focus of the interviews, many patients spoke about their experience of having a biopsy, which some described as very painful. One patient who struggled to tolerate this procedural pain questioned whether general anaesthetic could be used, and suggested he wanted better information to prepare himself.

#### Post-procedural pain

While receiving inpatient care post-TTP, Connie experienced intense pain during the first night. She neither expected nor was prepared for this. She was concerned that some staff lacked an awareness of the pain associated with thoracoscopy and suggested that specific pain-guidance should be available to both patients and staff focusing on post TTP management. Despite being unprepared, Connie felt that overall, her pain was well managed during her MPE care.

#### Drain removals and IPC complications

Intercostal drain removal was reported as being extremely painful and unexpected by one patient, contradicting what he had been led to believe.

Two patients randomised to receive TTP+IPC experienced pain stemming from complications with their IPC.

Francis had experienced severe pain during unsuccessful home fluid drainages by the district nurses. His wife Ruth explained the impact of this.

Joshua believed that his body was ‘rejecting’ the catheter due to intense pain which was not relieved by morphine.

### Preferences in MPE care

Patients’ preferences and views on MPE management, specifically around the option for home or hospital-based care, could be separated into views informed by experience and preconceived ideas that were sometimes based on misconceptions.

#### Preferences based on experiences of MPE management

Some participants exhibited a strong preference for outpatient care and a swift discharge from hospital, over hospital-based care. Reasons for this included a desire to be in familiar and comfortable surroundings, as well as for independence.

Keith was randomised to standard care but needed to have an IPC inserted subsequently: ‘The first one was an experiment to see if it would stay up stay stuck, but it didn’t, so I had the second. So, it was very tolerable and that’s what I’ve got now, the permanent one (…) It’s better out [of hospital] because I can maintain a life’ (Keith, patient, TTP).

Following a successful pleurodesis and subsequent IPC removal, Stephen also expressed a preference for outpatient care: ‘Great to be able to go home and be in my own bed (…) I’m tube-free!’ (Stephen, patient, TTP+IPC).

A preference for outpatient care was expressed by those who had a positive experience with the district nursing team. Stuart found drainage by district nurses acceptable, but he felt that over the longer-term this might become intrusive and favoured the idea of training carers to support IPC drainage.

In contrast, a few patients described a preference to be treated and managed in hospital due to their belief that it was safer, citing pain management as one of their reasons for this preference.

Some patients described negative aspects of home drainage in relation to the limited availability of supplies in the community and one patient described how having limited access to IPC drainage bottles made her feel undervalued due to her palliative status.

#### Speculative treatment preferences: perceptions of burden associated with IPC and combined procedure pathway

Patients preferences were based on their experience of treatment, as well as their preconceptions of the alternative treatment. Some patients described having negative preconceptions about the potential burden of IPC.

Arthur expressed relief that, despite being randomised to receive the TTP+IPC, he did not receieve an IPC because it was deemed clinically unsafe. He viewed the TTP+IPC randomisation arm as the ‘awkward pathway’*,* and was concerned that an IPC would place restrictions on his daily life.

In contrast, Connie (patient) was randomised to receive TTP only and described feeling initially disappointed not to have an IPC inserted. However, with hindsight, following her positive and successful experience of pleurodesis, she described being pleased with her randomisation and felt she had avoided the extra burden and resources associated with an IPC.

### Measuring MPE: rethinking patient relevance

#### Acceptability, relevance, and usability of PROMS

As part of the trial, patients were asked to complete data-capture diaries containing patient-reported outcome measures (PROMS) to record their experience of specified symptoms, healthcare interactions (frequency and reason for attendance) and fluid drainage (volume in mls). Patients were asked to record the presence and severity of breathlessness and chest pain over the first 4 weeks of the trial by drawing a static mark on a horizontal Visual Analogue Scale (VAS) (online supplemental appendix D). When asked specifically about the acceptability of the PROMS, most reported no significant problems.

While many patients appreciated the speed at which PROMs could be completed, others felt they lacked personal relevance, with one patient expressing concern that the measure did not capture symptom fluctuation. Additionally, some patients struggled to understand how to accurately complete it.

The patient diaries were found to be a useful measure for carers as they provided an accurate account of the patient’s health over time. This was reflected on positively by Emma (a carer), who felt that the diaries provided a helpful record for her to refer to, helping her to recognise and appreciate small moments of progress amidst the unfamiliar and potentially overwhelming context of this progressive disease.

## Discussion

To our knowledge, this is the first UK-wide, in-depth qualitative study of patients with MPE and their carers that examines personal experiences of differing management procedures and effusion control.

The over-arching finding of this study is that patients and their carers found the experience of the combined procedure tolerable and acceptable in the context of the TACTIC trial. Further analysis resulted in the development of four core themes which depicted the experience of MPE management: (1) making sense of MPE, (2) experiences of pain, (3) preferences in MPE care and (4) measuring MPE: rethinking patient relevance. Our findings characterise the patient and carer experiences of MPE management including their treatment expectations, preferences and challenges.

### Pain

A key finding was the experience of unanticipated periprocedural pain.

Examples of intraprocedural pain demonstrated here are in keeping with qualitative findings by Twose *et al*^6^ in which pain was noted in TT. This is also echoed by the TACTIC RCT findings, which demonstrated that instances of chest pain requiring analgesia were higher in patients receiving TTP+IPC (56%), in comparison to those who received TTP only (40%).^24^ While instances of pain may not be surprising to a clinical audience, our findings suggest that patients felt unprepared for the severity of pain they experienced through the management pathway including pain during chest drain removal. Pain following talc pleurodesis was also reported, echoing findings from the TIME 1 trial in which pain was found to last up to 12 hours post-procedure.^25^ However, it is important to recognise that pain was not experienced by all patients, and some commented on their relief at the lack of pain, for which there are many possible reasons. One reason for this may be regional differences in the conduct of medical thoracoscopy procedures. Multiple researchers have noted significant national variation of medical thoracoscopy practice, including differences in the types of sedation and analgesia used.^26–28^

Although there is a standard analgesic pathway, our findings further emphasise the importance of analgesia being offered to patients following thoracoscopy. Findings also suggest a need to re-examine how consistently analgesia is used, in order to understand how effectively acute pain is being managed in this population, nationally. Additionally, clear communication to emphasise the potential for pain and signposting during the consent process prior to medical thoracoscopy is necessary to ensure that patients feel adequately prepared for the procedure.

### Flexibility of care

Our analysis also demonstrated that care preferences were informed by individual circumstances and personal priorities. For those who desire it, IPC provides patients with an opportunity for increased independence in their MPE management. Offering training to carers and next of kin to drain an IPC in the community may provide an increased sense of freedom and flexibility, while minimising the burden of community drainage on nursing teams. This may be of particular relevance for patients whose IPC is in situ for longer. However, this approach warrants caution, as a key consideration of reduced oversight from district nurses, is the possible increased risk of infection. Adequate training could mitigate the increased infection risk; however, delivering this to a high standard consistently would have significant resource implications. Appropriate safety-netting measures, including regular review and clearly defined escalation pathways, would also need to be implemented.

Within our study, many of the patients randomised to receive an IPC had their devices removed prior to time of interview. Without data from those experiencing long-term IPC placement, our findings may lack insight into this experience. This may explain why the psychosocial impact of longer-term IPC placement^10 29^ was not reflected in our thematic analysis.

### Patient relevance

In viewing the experience of MPE through the lens of patients and those who care for them, new insights may be gleaned into the impact of different management pathways. Our findings support the arguments by Twose *et al*^6^ and build on a recent systematic review of patient reported outcomes in pleural effusion trials. Mishra *et al*^30^ emphasise the need for outcome measures that are reflective of patient priorities, validated for use with this patient group and responsive to pleural fluid drainage.

Our findings contribute to the growing body of evidence supporting a review of current MPE outcome measurements and the inclusion of a broader range of PROMs to generate a more nuanced understanding of how MPE management options affect patients’ everyday lives.

### Strengths and limitations

A strength of this study is the inclusion of patients from eight regionally diverse hospital sites across the UK. This provides confidence that a broad spectrum of experiences was captured and negates concerns of localised bias. An added strength is the participation of carers who provided valuable perspectives, including insights into patients’ post-discharge transition and their care in the community. There is a growing need to understand the support requirements and priorities of a patient population whose condition is more increasingly being managed at home. The burden of MPE is also likely to increase due to continual improvements in cancer care and subsequent increases in survival rates. The inclusion of both qualitative methodologists and clinical specialists within the research team is also a great strength of this study, adding rigour and credibility to our findings.^31 32^

Limitations are also recognised. There was a predominance of patients with malignant pleural mesothelioma within the sample. This is reflective of the patients seen in pleural clinics and representative of the UK population included in previous MPE trials. Although it is not the aim of qualitative research to achieve statistical representation, only 11 of the 32 participants were female, suggesting our findings may not capture the full diversity of the experiences of female patients. However, more female carers took part than male carers. Additionally, patients were recruited from a cohort of participants in an interventional trial and are unlikely to be representative of the wider MPE population who did not meet the eligibility criteria, including frailer and more comorbid patients.

## Conclusions and recommendations

This interview study has demonstrated that patients and their carers found TTP+IPC tolerable and acceptable in the context of the TACTIC trial. Our results, consistent with others in this field, further reinforce the need to examine how this palliative patient group can be provided with flexible management options wherever possible to facilitate end of life care in line with their priorities. Findings also highlight the need for patients and carers to receive adequate information and preparation prior to procedures including clear communication regarding the potential need for repeat pleural procedures for patients undergoing TTP without IPC.^24^ Increased consideration of pain management during MPE care is needed, as well as better management of patients’ expectations about pain.

We argue for a greater research focus on understanding patient preference in MPE management and examining how this can be effectively facilitated for this heterogeneous group. Further investigation to establish which MPE outcomes matter most to patients is required to support clinicians in their development of individualised management plans. To achieve this, we echo the sentiments of other researchers in this field^30 33 34^ who call for the development of validated MPE-specific PROMS and the need for a robust Core Outcome Set for use in the development and conduct of future MPE clinical trials.

## Supplementary material

10.1136/bmjresp-2026-004098 online supplemental file 1

10.1136/bmjresp-2026-004098 online supplemental file 2

10.1136/bmjresp-2026-004098 online supplemental file 3

10.1136/bmjresp-2026-004098 online supplemental file 4

## Data Availability

Data are available upon reasonable request.
