# Supplementary figures and images for: Qualitative insights into patient and carer perspectives of malignant pleural effusion management: an embedded study within the TACTIC trial
Source: BMJ Open Respir Res. 2026 Jul 27;13(1):e004098. doi: 10.1136/bmjresp-2026-004098 (PMC13409097; doi:10.1136/bmjresp-2026-004098)

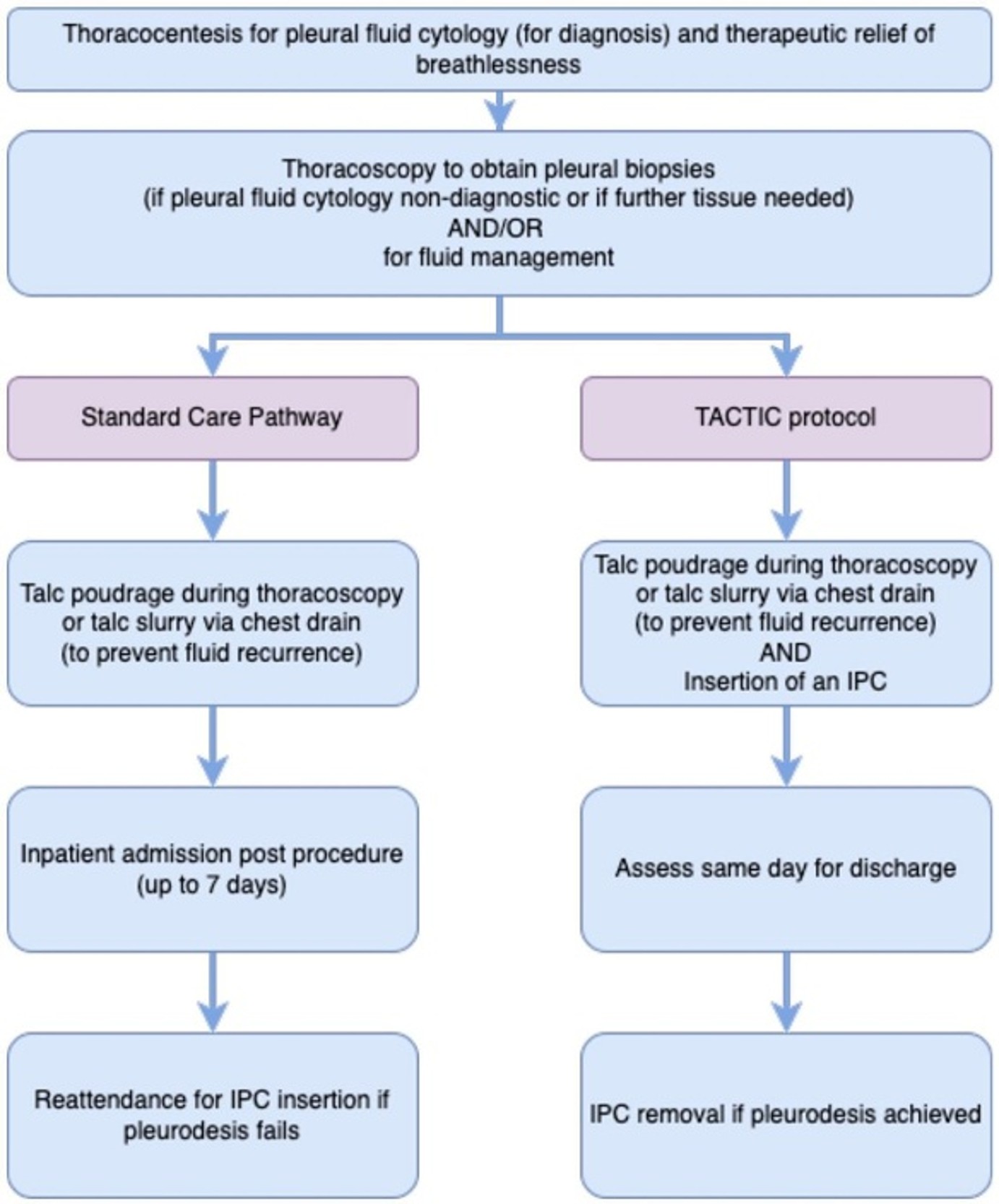

Supplement: online supplemental file 1 [file bmjresp-13-1-s001.jpg]

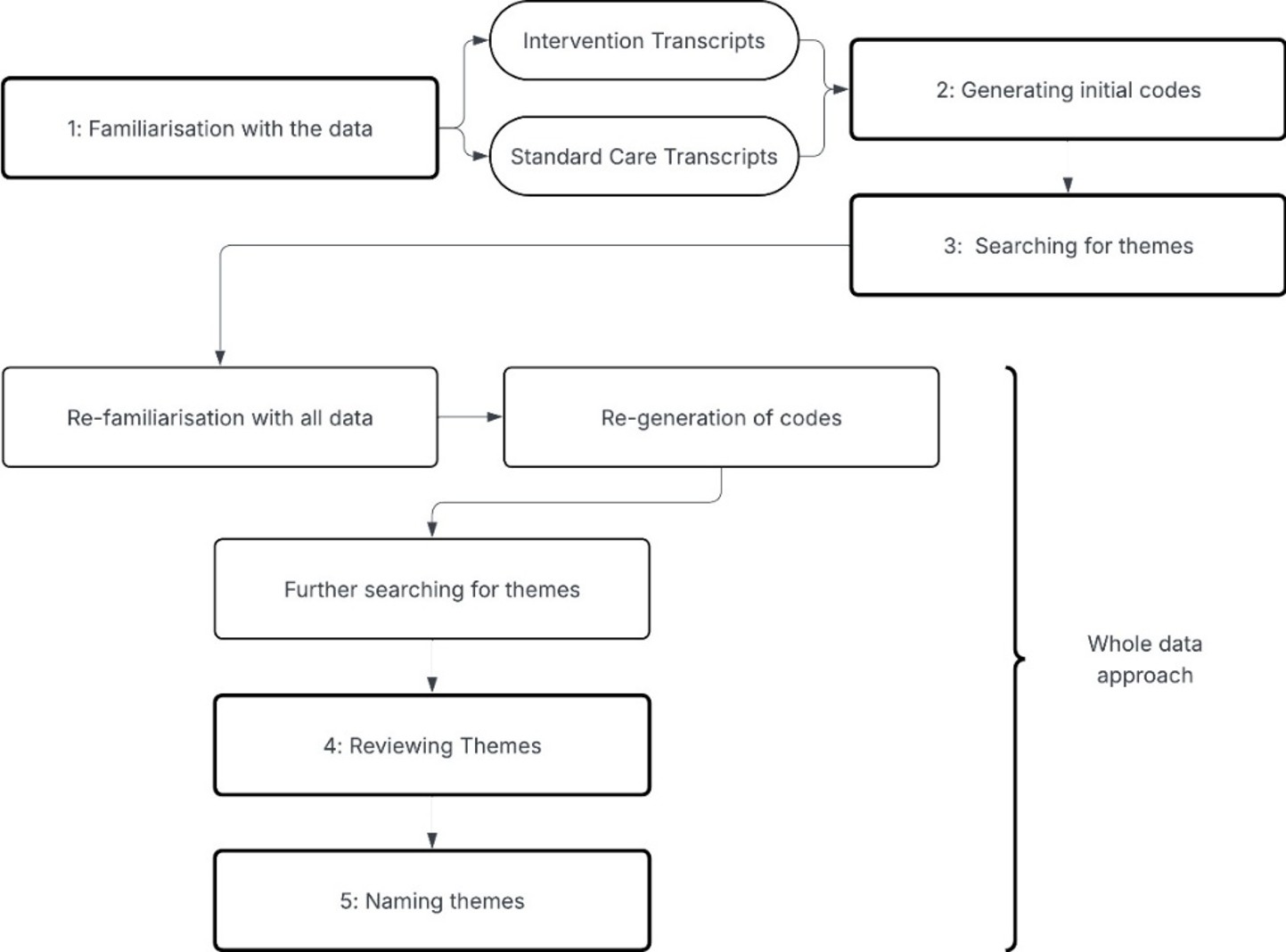

Supplement: online supplemental file 3 [file bmjresp-13-1-s003.jpg]

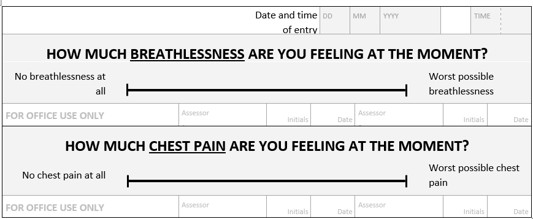

Supplement: online supplemental file 4 [file bmjresp-13-1-s004.jpg]
